# Atmospheric Degradation of Pure and Rosemary (*Rosmarinus officinalis* (L.)) Essential Oil-Added Antimicrobial Poly(Butylene Adipate-*co*-terephthalate) (PBAT) Films

**DOI:** 10.1021/acsomega.6c01636

**Published:** 2026-07-13

**Authors:** Chaianne K. S. Nascimento, Karlos D. E. Silva, Gabriel B. L. Vitorino, Amanda C. O. Diniz, Ivo D. L. Silva, Adriano N. Simões, Carlos A. Souza, Glória M. Vinhas, José F. Q. Pereira, Andréa M. S. S. Brito

**Affiliations:** † 28116 Universidade Federal de Pernambuco (UFPE), Department of Fundamental Chemistry (DQF), Professor Luiz Freire Avenue, ZIP code 50740-545 Recife, Pernambuco, Brazil; ‡ 67744 Universidade Federal Rural de Pernambuco (UFRPE), Academic Unit of Serra Talhada (UAST), Gregório Ferraz Nogueira Avenue, ZIP code 56909-535 Serra Talhada, Pernambuco, Brazil; § Universidade Federal de Pernambuco (UFPE), Postgraduate Program in Materials Science (PGMtr), Jornalista Aníbal Fernandes Avenue, ZIP code 50740-560 Recife, Pernambuco, Brazil; ∥ Universidade Federal Rural de Pernambuco (UFRPE), Academic Unit of Belo Jardim, PE 166, Km 03, ZIP code 55156-580 Belo Jardim, Pernambuco, Brazil; ⊥ Universidade Federal de Pernambuco (UFPE), Department of Chemical Engineering (DEQ), Professor Moraes Rego Avenue, ZIP code 50670-901 Recife, Pernambuco, Brazil

## Abstract

Most discarded packaging in the environment originates from petroleum-derived plastics, which are environmental pollutants. Many proposals for new packaging employ biodegradable materials; however, these materials often require additives to impart specific properties to the final products. Therefore, research related to degradation processes is necessary to ensure their environmental safety of them. The aim of this study was to investigate the degradation of pure poly­(butylene adipate-*co*-terephthalate) (PBAT) films and films added with 5% (w/w) Rosemary essential oil (REO) (*Rosmarinus officinalis L.*) under two different natural atmospheric conditions in a semiarid climate from northeastern Brazil. To this end, 66 rectangular samples with an area of 6.8 cm^2^ were exposed for 120 days under two conditions: condition (I) sunlight, wind, dust, humidity, and the presence of rain and condition (II) sunlight, wind, dust, humidity, and absence of rain. The samples were evaluated in terms of mass, thickness, solubility, opacity, visual appearance, and spectroscopic profile in the mid-infrared region using the carbonyl index (CI) and hydroxyl index (HI). An increase in solubility proportional to the water exposure time was also observed for both films, corroborating the visual analysis, which revealed the formation of fissures throughout the entire set of samples and a higher opacity in the oil-added films, especially under condition I. In addition, films exposed to condition I exhibited a greater mass loss than those under condition II. Differences in degradation between pure and REO-added films also became more evident, with a greater reduction in CI observed for the added samples under both conditions, indicating chemical degradation due to ester bond cleavage in PBAT. Principal component analysis indicated that up to 45 days, the samples exhibited high spectral similarity; however, thereafter, increasing discrimination between samples from the two experimental conditions was observed. This indicates that although the samples showed a similar degradation pattern over time, those exposed to condition I (with rain) were more strongly affected by climatic conditions and exhibited stronger evidence of chemical degradation.

## Graphical abstract

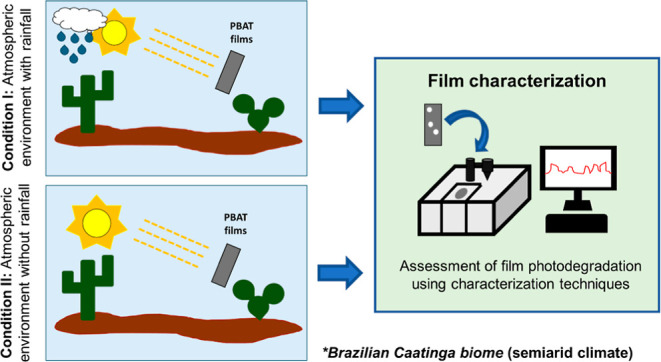

## 1 Introduction

The use of packaging for a wide range of purposes is common practice in modern society. In the food industry, packaging prevents direct contact between the product and the external environment, thereby promoting safe and high-quality consumption.^1^ According to the Brazilian Packaging Association,^2^ plastics rank first in packaging production, accounting for approximately 33.6% of this sector. Ahmed et al. associate the preference for this type of material with its versatility.^3^ Brazil is the fourth largest producer of plastic waste worldwide, yet only about 1% of this material is recycled.^4^

For this reason, there is global concern regarding the excessive amount of plastic-derived pollutants generated by the population, as highlighted in the 2023 report of the United Nations (UN).^5^ At the UN Environment Assembly held in 2022, the need to assess the plastic life cycle with the aim of eradicating this type of pollution was already discussed;^6^ however, as reported in the subsequent 2023 report, the trend in plastic production continues to increase. In view of this, the search for biodegradable materials as alternatives to conventional polymers has gained prominence.^7–9^ A polymer that has been identified in the literature as promising for packaging applications is poly­(butylene adipate-*co*-terephthalate) (PBAT), commercially known as Ecoflex. This polymer is obtained from aliphatic and aromatic monomers and exhibits good mechanical properties, in addition to being marketed as biodegradable.^10–12^ Kijchavengkul et al. emphasize that the biodegradable nature of this copolyester is attributed to the aliphatic segment present in its chemical structure, referred to as the butylene adipate group.^13^

Besides its biodegradability, which is essential for food packaging applications, it is crucial that the material be capable of preserving food quality by providing protection against environmental factors that may promote undesirable processes, such as oxidation or microbiological contamination.^14^ These properties can be imparted to the product when it is enclosed in active packaging, which contains additives with antimicrobial activity.^15–17^ Among the substances that exhibit such characteristics are essential oils (EOs).

Rosemary essential oil (REO) (*Rosmarinus officinalis*), for example, has been widely used in the literature as an additive in active packaging due to its antioxidant, antimicrobial, and aromatic properties.^18–20^ An example of the growing interest in the incorporation of EOs and their antimicrobial activity in food packaging is the study by Takala et al.,^21^ which evaluated antimicrobial activity against *Escherichia coli*, *Salmonella typhimurium*, and *Listeria monocytogenes* in fresh broccoli stored at 4 °C and packaged in methylcellulose (MC) films and polycaprolactone/alginate (PCL/ALG) blend films containing two formulations of EO mixtures: mixture A (organic acids + rosemary extract + EO of Asian spices) and mixture B (organic acids + rosemary extract + EO of Italian spices). The bioactive films proved effective in significantly reducing the growth of *L. monocytogenes* and *E. coli* over 4 days of storage. These findings reinforce the potential of such materials to act as natural antimicrobial barriers, releasing volatile compounds capable of inhibiting pathogens and contributing to the safety of fresh-cut vegetables.

Falcão et al. evaluated the permeability, biodegradation, and properties of PBAT through accelerated aging and artificial radiation.^22^ Barreto et al. also investigated the degradation of this polymer; however, in this case, its decomposition was assessed using aquatic microorganisms.^23^ More recently, Oliveira et al. analyzed the behavior of plastic bags made from a different polymer, low-density polyethylene (LDPE), in air, water, and soil.^24^

Thus, considering that studies such as that of Souza^20^ indicate the incorporation of EOs into polymeric films as promising active substances for obtaining packaging materials with antioxidant and antimicrobial properties, it is noteworthy that several studies have already evaluated the functional and physicochemical properties of REO as a potential active agent in different polymeric matrices for active packaging applications.^25–29^ However, when considering the natural atmospheric degradation of PBAT containing natural additives, the available results remain scarce, since most studies focus on soil biodegradation. In addition, previous studies^30–32^ evaluated the degradation of PBAT films containing clove and/or orange EOs under semiarid climatic atmospheric conditions and different additive concentrations. However, no study has monitored the degradation process during the same seasonal period, both in the presence and absence of rainfall, using the same group of samples.

Therefore, the main objective of this study was to investigate the degradation behavior of PBAT/REO films under different conditions (with and without rainfall) in a natural semiarid climate atmosphere (the Caatinga biome, which is exclusively Brazilian), using a 5% additive concentration in the PBAT matrix. It is noteworthy that, in previous studies, variations in the additive content incorporated into PBAT did not significantly affect the overall atmospheric degradation process.^30^ This evaluation was carried out using parameters such as mass loss, thickness, solubility, opacity, physical/visual appearance, and spectroscopic profile in the mid-infrared region (using the carbonyl index (CI) and hydroxyl index (HI)) as well as principal component analysis (PCA).

## 2 Methodology

### 2.1 Preparation of Polymeric Films

A total of 2.0000 g of poly­(butylene adipate-*co*-terephthalate) (PBAT) were weighed and dissolved in 50.00 mL of analytical-grade chloroform and then maintained in a closed system (a beaker covered with a watch glass) under constant stirring on a Fisatom magnetic stirrer for 120 min. The prepared solutions were transferred to Petri dishes with a diameter of 14 cm and kept in a fume hood for 48 h to allow for complete solvent evaporation. After this process, samples were removed from the Petri dish and maintained under a dry environment inside a desiccator.

For the films containing REO, the same methodology as described above was employed. REO was added at 5% (w/w) (0.1000 g of EO and 1.9000 g of PBAT) to the PBAT solutions that had already been under constant stirring for 120 min. After the addition of REO, the solutions were stirred for an additional 30 min before being subjected to the same solvent evaporation process as the pure films. The concentration of 5% (w/w) REO was used in this study based on the work of Andrade et al.,^33^ in which this was the lowest concentration evaluated and still showed significant antimicrobial activity in the PBAT/orange EO system. Although the REO concentration was selected based on previous studies reported in the literature rather than through an optimization procedure, this concentration was considered suitable for evaluating the effect of photodegradation on PBAT films.

The prepared films were cut into dimensions of 4.5 cm × 1.5 cm, resulting in 66 samples, which were exposed and analyzed with the more opaque surface facing upward in order to maximize the absorption of solar radiation as well as infrared and UV–vis radiation from the analytical equipment.

### 2.2 Determination of Film Mass, Thickness, and Visual Appearance

The average initial mass of each sample was determined prior to exposure by weighing the films in triplicate on an analytical balance. The average thickness of each film was also determined by measurements taken at three different points on each sample using a Mitutoyo disk micrometer with a measuring range of 0–25 mm and a precision of 0.001 mm. All samples were visually evaluated before and after exposure to solar radiation, and visual characteristics such as color, homogeneity, and brittleness were recorded.

Photographic records were obtained 48 h after sample collection to standardize the visual evaluation of the films. The images were acquired using a Moto G60 smartphone equipped with a 108 MP main camera, a high-resolution sensor, an f/1.9 aperture, and image capture capability with a good level of detail and visual fidelity under adequate lighting conditions.

### 2.3 Mass Loss Calculation

The percentage mass loss of the samples under study is calculated according to eq 1, where w_i_ is the initial mass and w_f_ is the final mass of the samples.^34^

1 $$Mass\;loss\;\left(\%\right)=\left(\frac{w_{i}-w_{f}}{w_{i}}\right)x100$$

### 2.4 Determination of Film Opacity and Solubility

The apparent opacity (absorbance units normalized by film thickness – au/mm) is determined using eq 2 based on the initial UV–vis data, where Abs_600_ is the absorbance at a wavelength of 600 nm (this wavelength was selected because it corresponds to the yellow region of the spectrum).^35^

$$Opacity\;\left(au\;{mm}^{-1}\right)=\frac{{Abs}_{600}}{Thickness\left(mm\right)}$$ 2

### 2.5 Solubility

Solubility was determined in authentic triplicate, in which each film was weighed (in triplicate) to obtain the average initial mass and then immersed in 80 mL of distilled water, remaining under constant stirring on a magnetic stirrer for 1 and 24 h, respectively. After each of these agitation periods, the film was placed in a Lucadema 161/01 BOD incubator at 40 °C for 24 h to allow water evaporation. Subsequently, the films were removed from the incubator and placed in a desiccator containing silica gel until they reached room temperature, and then weighed (also in triplicate). The percentage solubility in water (%) is calculated according to eq 3, where w_i_ is the initial weight and w_f_ is the final weight.^36^

3 $$Solubility\;\left(\%\right)=\left(\frac{w_{i}-w_{f}}{w_{i}}\right)x100$$

### 2.6 Natural Degradation Experiments

Two experimental conditions were established for the assays: condition I (effects of solar radiation, wind, dust, humidity, and rain) and condition II (effects of solar radiation, wind, dust, humidity, and absence of rain). Exposure of the films under natural atmospheric conditions was carried out in the Sertão do Pajeú region, Pernambuco, Brazil.

The samples were placed on adapted trays with perforations to prevent water accumulation on the surface and were tied on a support made of PET bottle parts using a nylon (polyamide) thread to avoid being carried by the wind action. Along degradation period, all parts that could be recovered were analyzed. The trays were positioned on a wooden support with an approximate inclination of 45°. A total of 33 pure PBAT samples and 33 PBAT samples containing rosemary oil were prepared, from which one sample of each type was set aside to serve as a reference (day zero).

The selected sites exhibited no shading throughout the day. Samples that remained in Serra Talhada, PE, were subjected to sunlight and rainfall conditions (condition I).^31^ Half of the total samples were separated and transported for exposure in Custódia, PE, where they were subjected only to solar radiation effects (condition II).^32^ Every 15 days, two samples of each film type were removed for analysis, completing a total of eight cycles over 120 days.

### 2.7 Fourier Transform Infrared Spectroscopy

Before film exposure, their respective spectra in the mid-infrared region were acquired using a PerkinElmer Frontier FTIR spectrophotometer equipped with a universal attenuated total reflection (UATR) accessory. The operating range was 600–3600 cm^–1^, with a resolution of 8 cm^–1^ and 8 scans, using air as the background.

The samples were also analyzed in the UV–vis region using a PerkinElmer Lambda 25 spectrophotometer over a wavelength range of 200–700 nm. FTIR and UV–vis analyses were performed in triplicate, and the data were averaged. All spectroscopic analyses were carried out on the surface of the samples that were exposed to solar radiation.

The carbonyl index was calculated from the FTIR spectra according to eq 4, where CI is the carbonyl index, Abs_C=O_ is the absorbance variation of the ester carbonyl peak (1712 cm^–1^), and Abs_C–H_ is the variation of the reference absorbance (726 cm^–1^), relative to a baseline point, following the methodology adapted from Baumhardt-Neto and De Paoli.^37^

$$IC=\frac{{Abs}_{C=O}}{{Abs}_{C-H}}$$ 4

The hydroxyl index of the pure films and those added with REO was calculated from the FTIR spectra obtained for each predetermined exposure period, according to eq 5. In this equation, HI is the hydroxyl index, Abs_O–H_ is the absorbance variation of the hydroxyl band (3100 cm^–1^), and Abs_C–H_ is the absorbance variation selected as the reference (726 cm^–1^), relative to a baseline point, following the methodology adapted from Fechine et al.^38^

5 $$IH=\frac{{Abs}_{O-H}}{{Abs}_{C-H}}$$

### 2.8 Meteorological Data Record

During the study, atmospheric condition data, including temperature, precipitation, relative humidity, and mean global radiation, were recorded and monitored with the A349/INMET weather station.

### 2.9 Data Treatment

. All infrared data were preprocessed and analyzed using the PLS Toolbox package (Eigenvector Research, Inc., PLS_Toolbox, Version 8.6) within the MATLAB software environment (R2024b.441.243.592, MathWorks). The results for all parameters were evaluated by comparing their average values with a *t*-test.

## 3 Results and Discussion

### 3.1 Visual Appearance and Opacity of the Films

The prepared films showed no bubbles; however, most of them were not uniform, with one side being thicker than the other, which hindered cutting. Both the pure PBAT film and the REO-added film exhibited one shinier surface (in direct contact with the Petri dish) and one opaque surface (in contact with air). Both films displayed a whitish coloration, and the rosemary-added films exhibited a strong characteristic odor.

Figure 1 shows the fragmentation stage of the samples exposed to conditions I and II, with changes in coloration and dust accumulation being observed (especially under condition I) as a result of weathering. Figures 1a,b correspond to samples exposed to sunlight and rain (condition I), whereas Figures 1c,d correspond to samples exposed only to sunlight (condition II).

**Figure 1:**
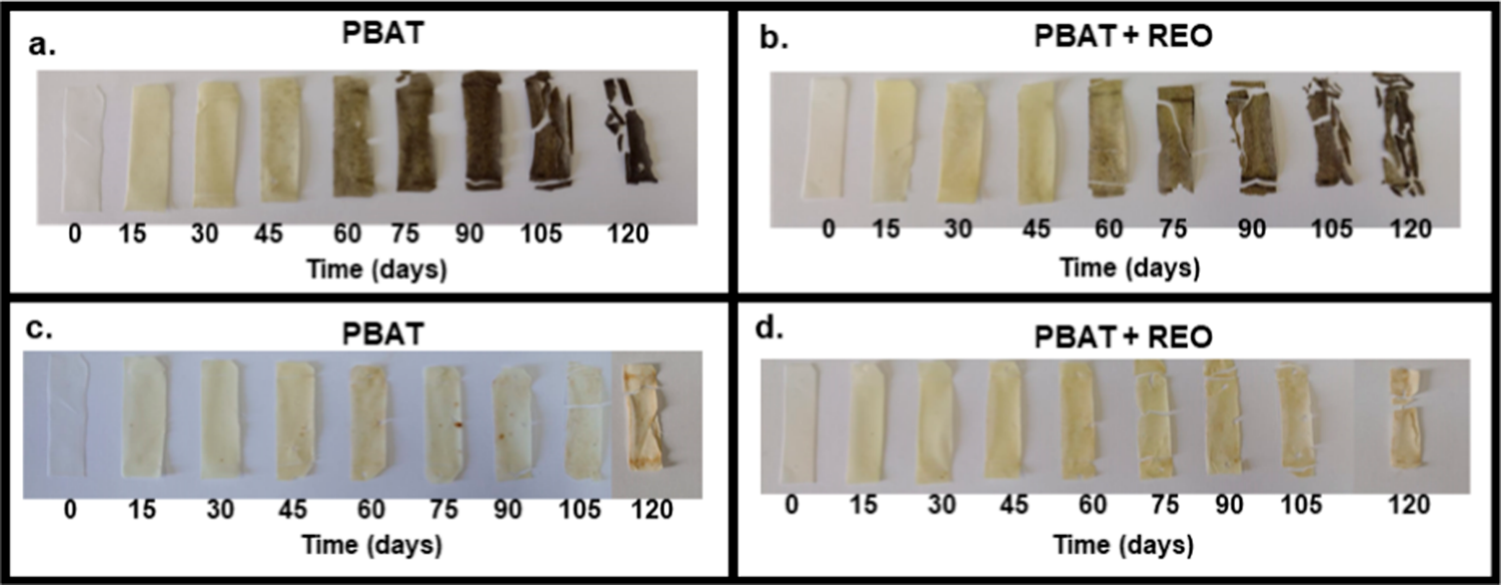
Visual analysis of the films: (a,b) under sunlight and rain (condition I) and (c,d) exposed only to sunlight (condition II).

Samples exposed only to sunlight exhibited fragmentation but to a lesser extent than those under condition I, as also discussed in the mass loss analysis. Up to 45 days, no significant fragmentation or cracking was observed; after 60 days, the samples lost their structural integrity and were fragmented into small pieces. Over time, the samples became heavily soiled and fragmented (especially under condition I), which hindered proper contact with the ATR crystal during spectroscopic analyses, resulting in spectra with increased radiation scattering.

The phenolic compounds present in REO can absorb UV radiation, preventing or reducing the passage of this radiation through the film.^39^ Depending on its origin, REO may contain terpenoids, sesquiterpenoids, and phenolic compounds, with hydroxyl, carbonyl, and epoxy groups that can react with the hydroxyl and carboxyl groups of PBAT, forming longer polymeric structures and thus reducing film degradation, as observed in condition II (only under sun exposure).^40–42^

As will be seen later, only a proportion of 5% rosemary oil was used in the total mass of the PBAT film, and therefore, it was not possible to verify the molecular interaction process via infrared, since the characteristic bands overlap and the peaks corresponding to the functional groups of the components present in the REO were not observed.

The average mass (g) and thickness (mm) of pure PBAT and PBAT containing REO ranged from 0.0499 to 0.1767 g and 0.0566 to 0.1944 g, respectively, while thickness varied from 0.10 to 0.24 mm for pure PBAT and from 0.10 to 0.30 mm for PBAT with REO. Thicker films (average thickness of 0.22 mm for pure PBAT and 0.20 mm for PBAT with rosemary) were selected for exposure to sun and rain conditions to allow for extended observation periods. Films added with REO exhibited slightly higher average opacity compared to pure PBAT films. From a food packaging perspective, it is important that the material protect the product from the effects of light, a property that may be directly related to coating opacity. Regarding material degradation, this opacity may also play a role, since, according to Mena et al.,^43^ photodegradation requires the transmission of light through the polymer surface. Considering that the average opacity of the added films was slightly higher than that of the pure PBAT films, the added films presented an average value of 15.1 au ·mm^–1^ , whereas the pure films exhibited an average value of 14.8 au· mm^–1^. However, considering 95% of confidence, the difference between these means is not significant, and consequently the opacity difference between than was also not significant.

### 3.2 Mass Loss

Figure 2 shows the mass loss of the samples under the two investigated conditions over 120 days of exposure. Figure 2a presents the results for samples exposed to sunlight and rain (condition I), whereas Figure 2b shows the mass loss of samples subjected only to solar radiation (condition II).

**Figure 2:**
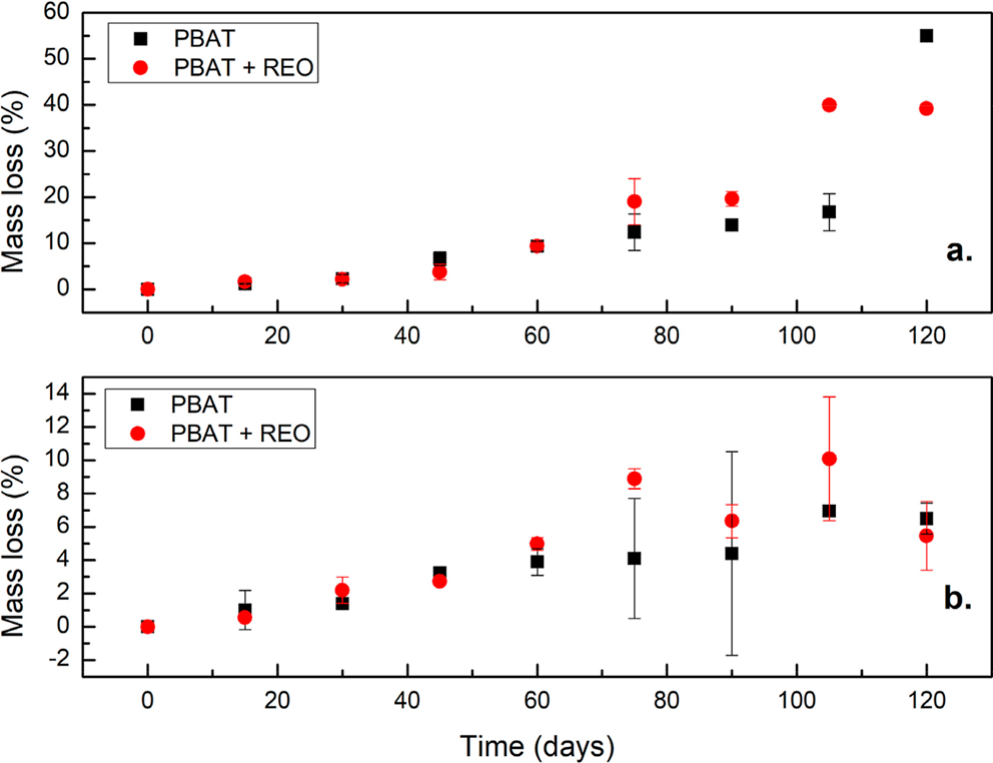
Mass loss of the films: (a) under all atmospheric natural (condition I) and (b) exposed under all atmospheric conditions except rain (condition II).

It can be observed that the samples simultaneously exposed to sunlight and rain exhibited greater mass loss than those subjected to only solar radiation, which corroborates the discussion presented in the solubility section, where the presence of water contributes to increased material wear. Under both conditions, the pure films showed greater mass loss at the end of the 120 day period (approximately 54.9% under sunlight and rain; 6.49% under sunlight) compared with the films containing REO (approximately 39.2% under sunlight and rain; 5.46% under sunlight).

However, because of the high standard deviation and variations in environmental conditions, such as high rainfall indices (for example, a total of 157.1 mm in January 2022, according to IPA^44^), slight differences in the values can be observed. The wind also affected the mass loss, as some samples were partially or fully carried away, especially after 75 days of exposure.

The results of this study are consistent with the findings discussed by Falcão et al.,^22^ who reported degradation ranging from 49 to 62% when artificial UV radiation was applied. However, the mass loss observed in the present work is lower than that reported by those authors, which can be attributed to the fact that this study was conducted under natural environmental conditions.

### 3.3 Solubility

Although PBAT is known to exhibit hydrophobic characteristics,^45^ this solubility analysis was performed to evaluate the behavior of pure and rosemary oil-added PBAT films in the presence of water, since the samples were exposed to rainfall under condition I. It can be observed that the solubility of both the pure material and that incorporated with rosemary oil increased with water exposure time, reaching approximately 1.15% for PBAT with rosemary oil and 0.98% for pure PBAT after 24 h of immersion in water. Although the difference in the mean solubility values between the pure films and those incorporated with REO was small, the films containing REO exhibited slightly higher solubility compared to the pure films, which is consistent with findings commonly reported in the literature. In general, according to the considerations of Brandelero, Grossmann, and Yamashita,^46^ the incorporation of oil tends to reduce the hydrophilicity of polymers. Nevertheless, even with low solubility percentages, water (originating from rainfall to which the samples under condition I were exposed) increased film wear, leading to greater fragmentation of the samples.

### 3.4 Infrared Spectroscopy (FTIR)

Through these analyses, performed prior to film exposure, it was possible to obtain the main absorption bands of the pure PBAT films and the films added with REO, as well as the spectrum of REO itself, as shown in Figure 3. The most intense peak at 1712 cm^–1^ corresponds to the C = O carbonyl groups of ester moieties, which are an important feature in the evaluation of polymer degradation.

**Figure 3:**
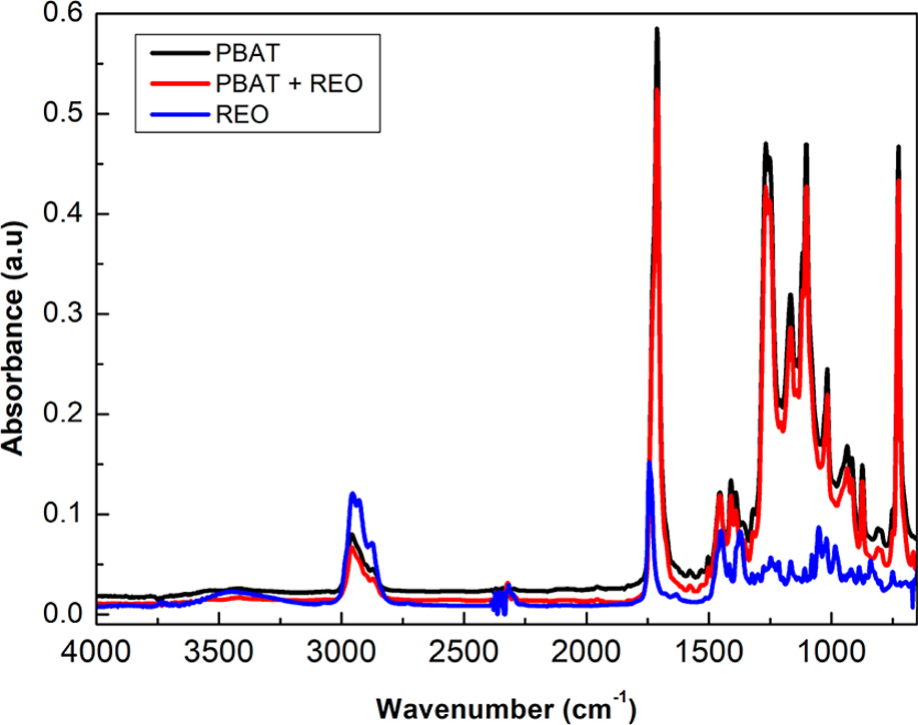
FTIR spectra of REO, pure PBAT, and PBAT added with REO.

According to Lee et al., the constituents of REO may vary depending on cultivation conditions;^47^ however, Alikooyi et al.^48^ and Xia et al.^49^ emphasize that it is predominantly composed of 1,8-cineole (eucalyptol), α-pinene, and camphor.

Observing the behavior of the films incorporated with REO, it can be noted that most absorption bands overlap, coinciding at the same positions. By acquiring FTIR spectra of the films before and after exposure, it was possible to evaluate the effects of photodegradation on their structure through a comparison of the average absorption bands according to the specific exposure times (0 and 120 days).

In Figure 4, a decrease in the absorption intensity of the bands can be observed, especially for samples exposed to all regional climatic stressors in both pure and oil-added films. This behavior corroborates the discussions presented by Moraes Filho,^50^ who reported similar findings. However, no noticeable shifts in these bands were observed.

**Figure 4:**
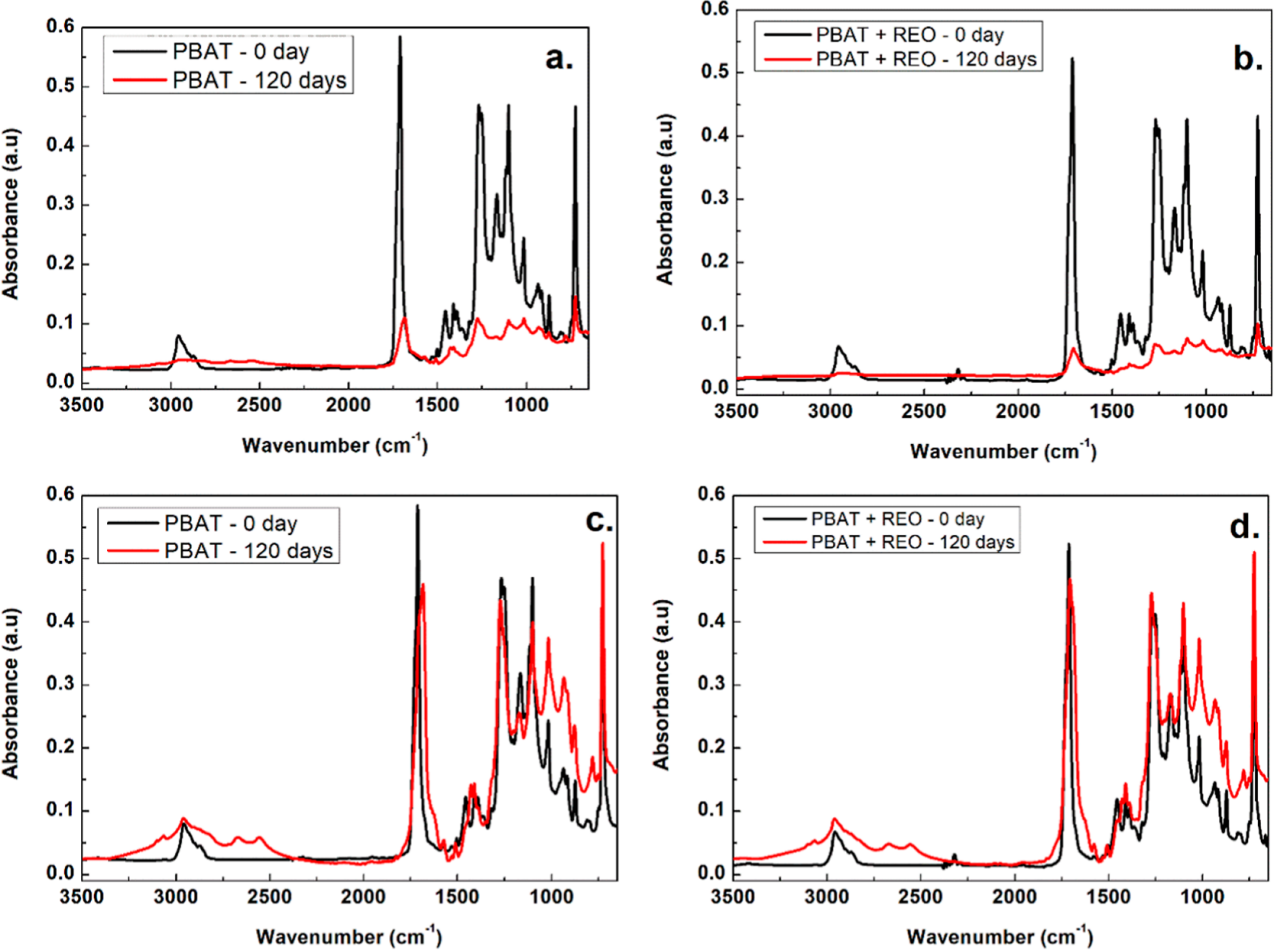
Normalized FTIR spectra of the films at 0 and 120 days of the biodegradation test: (a,b) condition I and (c,d) condition II.

To better visualize the structural changes in the films over the degradation period, the carbonyl and hydroxyl indices were calculated. Since ester hydrolysis generates – OH groups, as discussed by Allen^51^ and Moraes Filho,^50^ the number of these groups in the molecules varies accordingly.

#### 3.4.1 Carbonyl Index

A decrease in the carbonyl index (CI) of both films was observed under both conditions (Figure 5a,b). However, under condition II, greater fluctuations in the CI (increases and decreases) were noted, as also reported in Moraes Filho^50^ biodegradation studies. This behavior can be attributed to the stage of material degradation as well as environmental variations.

**Figure 5:**
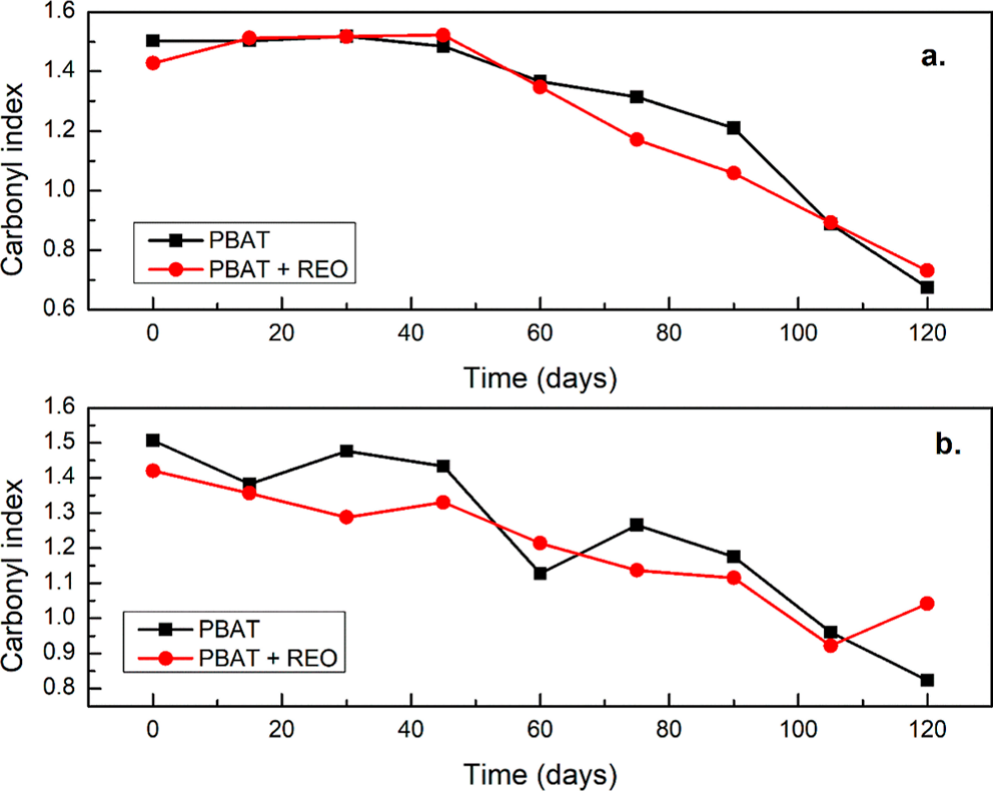
Carbonyl index for pure PBAT puro and PBAT added with REO under (a) condition I and (b) condition II.

In both cases, the CI of the pure films decreased slightly more than that of the added films, which is consistent with Souza et al.,^52^ where the pure film exhibited a greater reduction in CI. The differences in CI values between the pure and additive-containing films were not different up to 105 days of observation under either condition. However, at 120 days, the atypical variation observed in the CI of the additive-containing film resulted in a significant difference. When comparing the two exposure conditions, only the additive-containing films exhibited a significantly different reduction in CI, with a decrease of nearly 5% after 105 days of exposure. At 120 days, this comparison was not performed for the additive-containing films. However, the films containing REO also showed changes in their CI, indicating that they too underwent structural alterations, since, according to Moraes Filho,^50^ a static value would suggest minimal molecular change and, consequently, little to no material degradation.

In addition to structural changes involving the C=O bond, as described by De Paoli,^53^ who notes that photochemical degradation in polymers is initiated through this functional group, the formation of the hydroxyl band and the hydroxyl index are other relevant aspects that can be observed.

#### 3.4.2 Hydroxyl Index

Figure 6a,b shows the hydroxyl index (HI) for conditions I and II, which was calculated due to a slight tendency for the formation of the hydroxyl (−OH) band at 3100 cm^–1^. In both cases, the films exhibiting the highest HI after 120 days of exposure were the pure films.

**Figure 6:**
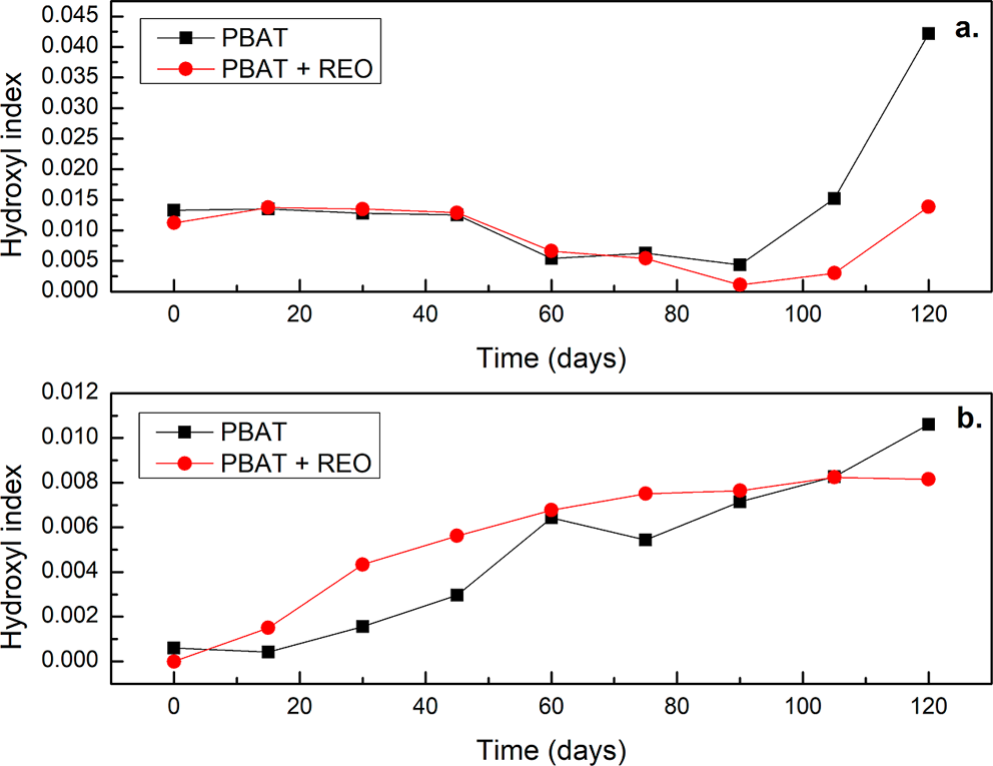
Hydroxyl index of pure PBAT and PBAT with rosemary essential oil: (a) condition I and (b) condition II.

The increasing HI indicates the degradation of the films, as the formation of – OH groups occurs due to the interaction and breakdown of the materials with the environment. This observation aligns with the discussions of Moraes Filho,^50^ who also reported a rising HI profile in biodegradation studies. It is important to note that the increase in HI is related to a decrease in CI, but not on the same scale. The chemical mechanism for photodegradation is described in more detail by Vitorino et al.^31^ The CI presented a stronger reduction after 40 days of exposure, indicating an acceleration in the degradation process by the reduction of carbonyl groups. After 180 days of exposure, the CI showed an anomalous increase; however, the HI maintained the expected increasing trend due to the presence of free radical reactions.

#### 3.4.33.4.3 Principal Component Analysis

The infrared spectra (Figure 4) showed a more informative region between 1800 and 700 cm^–1^, which was selected for PCA. As is common for samples analyzed at different time points, the spectra exhibited scattering effects, which were corrected through normalization to the 726 cm^–1^ peak and standard normal variate prior to constructing the PCA model.^54^

As shown in the score plot in Figure 7a, samples exposed to different climatic conditions initially exhibited similar behavior, clustering together up to approximately 45 days. After this period, the samples began to disperse along PC1 according to exposure time and along PC2 according to the climatic condition. The major variance (PC1 with 75.33%) is more related to exposure time for both experimental conditions. However, around 20% of the variance (PC2+PC3) explained the differences between experimental conditions, since a trend of separations between samples from these conditions is observed along the exposure time.

**Figure 7:**
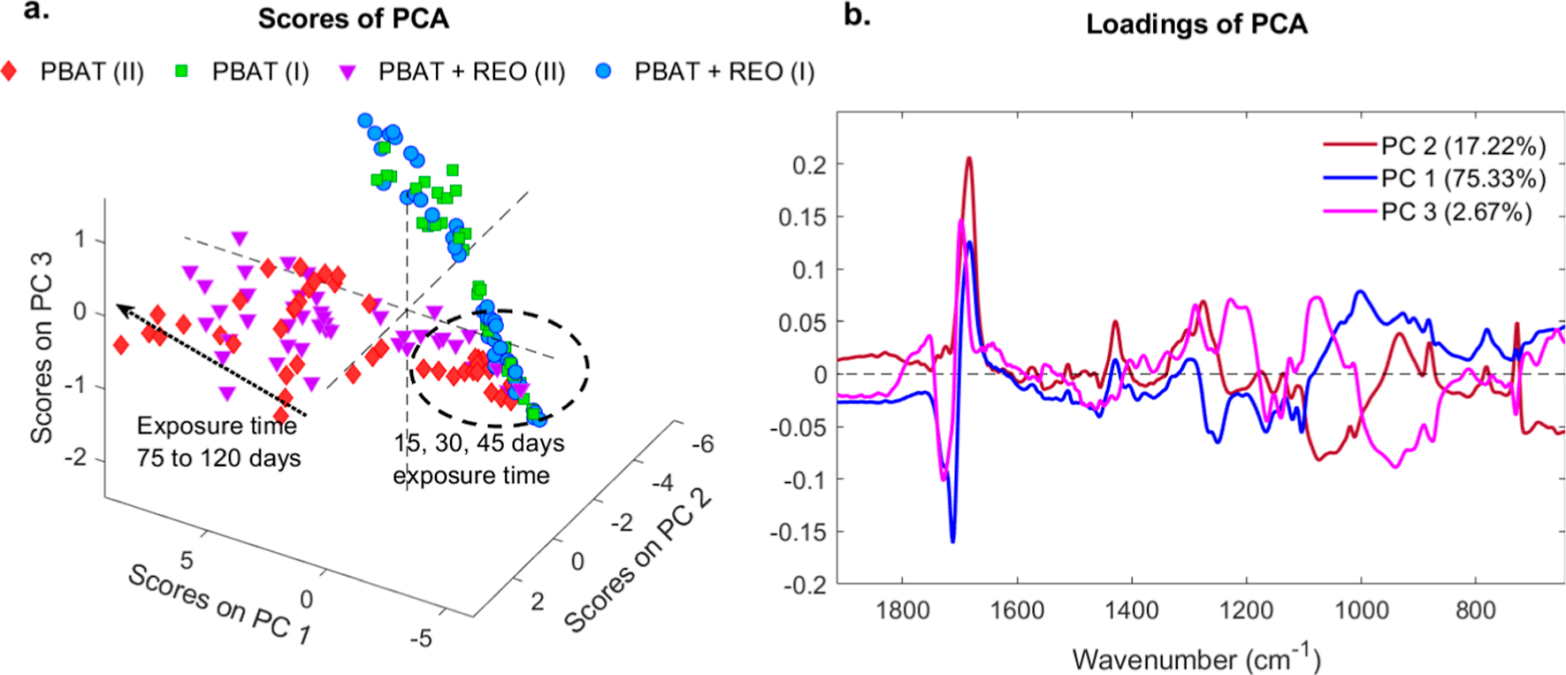
Scores (a) and loadings. (b) Plots of the PCA for pure PBAT and PBAT films added with rosemary essential oil (PBAT + REO) under exposure conditions I and II.

According to the loadings plot, the main spectral variables contributing to the dispersion of samples with exposure time are between 1650 and 1750 cm^–1^ (Figure 7b), presented in loadings of PC1 and corresponding to the modification of the ester carbonyl (C = O) band to caraboxylic acid carbonyl, in accordance with the Norrish II reaction described by Vitorino et al.^31^ and Majhi.^55^ The PC1 scores showed a trend of separation of samples according to their exposure time (Figure 7a). The PC2 scores, on the other hand, are primarily influenced by the carboxylic acid carbonyl band around 1700 cm^–1^ (Figure 7b), which positively affects both pure and oil-added PBAT samples exposed for at least 75 days under condition II (without rain). The loadings around 1020–1100 cm^–1^ (Figure 7b), typical of C–O banding bonds, presented a negative influence in the scores on PC2, being more related with samples from condition I. It means that the remaining positive loading bands are more related to the samples under condition II according to PC2.

In the present study, more pronounced degradation of pure PBAT was observed in the environment with sunlight and rain (condition I). PBAT degradation can occur through two main pathways: in a natural environment via enzymatic processes mediated by microorganisms, or nonenzymatically, with thermal degradation, photolysis, and chemical hydrolysis being the primary factors.^56–58^ Additionally, in a previous paper from our research group,^30^ samples were exposed to 12 h of sunlight, avoiding rain and night dew. Although the samples were prepared differently and the experimental approaches varied, the results were consistent: samples without water required a longer time to initiate the degradation process.

In light of these results, complementary analyses may contribute to a broader understanding of the behavior of the films during the degradation process. In this context, the investigation of physicochemical properties (mechanical and thermal properties), morphological characteristics (SEM), and functional properties (antioxidant and antimicrobial activity), involving both packaging performance and the photodegradation process, may be included in future studies to provide a more comprehensive understanding of the behavior of PBAT/REO films. However, the characterizations performed in the present study were sufficient to provide an adequate understanding of the degradation process of PBAT/REO films, allowing for the evaluation of the main structural changes in samples exposed and not exposed to rainfall.

### 3.5 Climatic Conditions Data

During the study period, the recorded maximum and minimum temperatures were 38.1 and 18.1 °C, respectively. Air humidity is an atmospheric factor that influences both temperature and rainfall, thereby contributing to the environmental degradation process. According to INMET,^59^ the maximum relative humidity recorded during the monitoring period was approximately 84%, while the minimum was 42.58%.

According to Rivaton and Gardette,^60^ and Hamid, Maadmah, and Amin,^61^ chromophore groups in aromatic polyesters such as PBAT absorb UV radiation between 300 and 350 nm, with maximum sensitivity at 325 nm, corresponding to the ester group. This makes it a key spectral component responsible for polymer degradation. Consequently, the average global radiation exposure in the environment during the study period was approximately 1757 kJ·m^–2^·h^–1^ (INMET, 2022),^54^ equivalent to an average incident energy of about 488.11 W·m^–2^ on the samples.

Regarding the rainfall index, fluctuations in precipitation were observed in the region; however, during periods of consistent rainfall, greater apparent wear was noted in the samples, as can be seen in Figure 1, which shows the stage of fragmentation of the samples exposed under conditions I and II. A change in color and accumulation of dust (especially under condition I) can be observed, resulting from the climatic stressors to which the films were exposed.

The PCA also revealed an increasing discrimination between the samples from the two experimental conditions, indicating that rainfall is an important variable in the degradation process. As shown in Figure 1, the samples exhibited a similar degradation pattern over time up to approximately 45–60 days of exposure. However, samples exposed to condition I (with rainfall) were slightly more affected by climatic conditions and showed more pronounced signs of chemical and physical degradation.

The samples exposed only to sunlight also exhibited fragmentation. However, as discussed in the mass loss section, it can be observed that these samples showed a lower degree of fragmentation and wear compared with those under condition I.

## 4 Conclusion

Exposure of the samples to atmospheric factors caused a marked increase in cracks, which was more pronounced in the pure PBAT samples. An increase in solubility was also observed depending on the duration of water exposure for both types of films, and regarding opacity, films incorporated with REO exhibited a slightly higher average value. Over the 120 day exposure period, samples under condition I lost more mass than those under condition II. Concerning the use of the additive, pure films lost more mass than films containing REO across all of the conditions studied. Through the decrease in the CI and the slight increase in the HI, it was possible to detect structural changes in the polymer chains of the films over the exposure period. The CI indicated that pure PBAT showed a greater tendency toward degradation compared to that of PBAT with the additive. Supporting these results, PCA provided a clearer understanding that the degradation of the samples is strongly dependent on the presence of water, which promotes fragility and material breakdown. PCA also revealed that the impact of water intensifies after approximately 60 days of exposure. Therefore, the present study proved highly relevant for evaluating the degradation of PBAT films with and without additives, with the overall results indicating that pure PBAT exhibited a greater tendency to degrade compared to that of PBAT incorporated with REO.
